# Long Noncoding RNA Expression Profiles of Lung Adenocarcinoma Ascertained by Microarray Analysis

**DOI:** 10.1371/journal.pone.0104044

**Published:** 2014-08-04

**Authors:** Gang Xu, Jie Chen, Qinshi Pan, Keta Huang, Jingye Pan, Wenhui Zhang, Jian Chen, Fangyou Yu, Tieli Zhou, Yumin Wang

**Affiliations:** 1 Department of Laboratory Medicine, the First Affiliated Hospital of Wenzhou Medical University, Wenzhou, China; 2 Department of Pathology, the First Affiliated Hospital of Wenzhou Medical University, Wenzhou, China; 3 Department of Intensive Care Unit, the First Affiliated Hospital of Wenzhou Medical University, Wenzhou, China; Barts & The London School of Medicine and Dentistry, Queen Mary University of London, United Kingdom

## Abstract

**Background:**

Long noncoding RNAs (lncRNAs) have been shown to be involved in the development and progression of lung cancer. However, the roles of lncRNAs in lung cancer are not well understood.

**Methodology/Principal Findings:**

We used a high-throughput microarray to compare the lncRNA and messenger RNA (mRNA) expression profiles in lung adenocarcinoma and normal tissue (NT) samples. Several candidate adenocarcinoma-associated lncRNAs were verified by real-time quantitative reverse transcription polymerase chain reaction (PCR) analysis. Using abundant and varied probes, we were able to assess 30,586 lncRNAs and 26,109 mRNAs in our microarray. We found that 2,420 lncRNAs and 1,109 mRNAs were differentially expressed (≥2-fold change) in lung adenocarcinoma samples and NT, indicating that many lncRNAs were significantly upregulated or downregulated in lung adenocarcinoma. We also found, via quantitative PCR, that 19 lncRNAs were aberrantly expressed in lung adenocarcinoma compared with matched histologically normal lung tissues. Among these, LOC100132354 and RPLP0P2 were the most aberrantly expressed lncRNAs, as estimated by quantitative PCR in 100 pairs of lung adenocarcinoma and NT samples.

**Conclusions/Significance:**

Our study ascertained the expression patterns of lncRNAs in lung adenocarcinoma by microarray. The results revealed that many lncRNAs were differentially expressed in lung adenocarcinoma tissues and NT, suggesting that they may play a key role in tumor development.

## Introduction

Lung cancer has the highest mortality rate of all cancers, and its incidence is gradually growing [1]. Non-small cell lung cancer (NSCLC) is the most common type of lung cancer (accounting for 80% of all cases) and includes squamous cell carcinoma, adenocarcinoma, and large cell carcinoma. Even though surgical resection, radiation therapy, and chemotherapy technologies are continuously improving, patients with lung cancer remain exceedingly vulnerable to relapse and death [2]. The cure rate for lung cancer is low and the average 5-year survival is less than 15% [3]–[6]. In recent years, a growing proportion of lung adenocarcinomas have been diagnosed as NSCLC that are attributable to causes such as environmental pollution. However, the mechanisms underlying lung cancer development have not been elucidated; thus, the study of lung cancer remains extremely important.

Long noncoding RNAs (lncRNAs) are transcript RNA molecules longer than 200 nucleotides that do not encode a protein and reside in the nucleus or cytoplasm [7]. LncRNAs are usually divided into five categories: sense, antisense, bidirectional, intronic, and intergenic. In recent years, a large number of lncRNAs have been identified, prompting the creation of the human lncRNA database, which provides lncRNA expression profiles and other important information [8]. The abnormal expression of lncRNAs has been implicated in a range of diseases, including cancer [9].

Studies have shown that lncRNAs known to be differentially expressed in normal cells and tumor cells are important for the regulation of gene expression; therefore, aberrant expression of lncRNAs can lead to abnormalities of gene expression and tumorigenesis. The altered expression of lncRNAs is a feature of many types of cancers and has been shown to promote the development, invasion, and metastasis of tumors by a variety of mechanisms [9], [10]. LncRNAs regulate expression on the epigenetic, transcriptional, and post-transcriptional levels [11]–[13].

LncRNAs have been shown to be involved in the development and progression of lung cancer; however, research concerning lncRNA involvement in lung cancer is in its infancy. Known lung cancer-associated lncRNAs are few and include HOTAIR, H19, ANRIL, MALAT1 (lung adenocarcinoma associated transcript 1) [14], [15], SCAL1 (smoke and cancer-related long-chain non-coding RNA1) [16], lncRNA AK126698 [17], and lncRNA GAS6-AS1 (GAS6 antisense RNA1) [18]. The identification of additional lung cancer-associated lncRNAs and their mechanism of action require further investigation.

In this study, we analyzed the expression patterns of lncRNAs and mRNAs in lung adenocarcinoma samples and compared them with the corresponding patterns in adjacent nontumorous tissue (NT) samples. Several of the differentially expressed lncRNAs were evaluated by SYBR RT-PCR in 100 pairs of tissue samples. Our results suggest that lncRNA expression patterns may provide new molecular biomarkers for the diagnosis of lung adenocarcinoma.

## Materials and Methods

### Patient samples

The lung adenocarcinoma samples and corresponding NT samples were prospectively collected from 105 patients of the First Affiliated Hospital of Wenzhou Medical University, China, from April 2012 to August 2013. Of these patients, five (the basic medical records see Table S1 in File S1) were used for microarray analysis of lncRNAs and 100 were used for additional evaluations (Table S2 in File S1). The diagnosis of adenocarcinoma was confirmed by histopathology. The lung adenocarcinoma and matched NT samples were snap-frozen in liquid nitrogen immediately after resection. This study was approved by the Institutional Ethics Review Board of the First Affiliated Hospital of Wenzhou Medical University, and all patients provided written informed consent for this study.

### RNA extraction

Lung adenocarcinoma cells were obtained by laser microdissection; the proportion of cancer cells in the tissue sections was 100%. We combined the five lung adenocarcinoma samples and the five corresponding NT normal samples, and the two grouped samples were subjected to RNA extraction. Total RNA was extracted using Trizol reagent (Invitrogen, Carlsbad, CA, USA), according to the manufacturer’s protocol. The integrity of the RNA was assessed by electrophoresis on a denaturing agarose gel. A NanoDrop ND-1000 spectrophotometer was used for the accurate measurement of RNA concentration (OD_260_), protein contamination (OD_260_/OD_280_ ratio), and organic compound contamination (OD_260_/OD_230_ ratio).

### Microarray and computational analysis

For microarray analysis, an Agilent Array platform (Agilent Technologies, Santa Clara, CA, USA) was employed. Sample preparation and microarray hybridization were performed based on the manufacturer’s standard protocols with minor modifications. Briefly, mRNA was purified from total RNA after removal of rRNA by using an mRNA-ONLY Eukaryotic mRNA Isolation Kit (Epicentre Biotechnologies, USA). Then, each sample was amplified and transcribed into fluorescent cRNA along the entire length of the transcripts without 3′ bias by using a random priming method. The labeled cRNAs were hybridized onto a Human LncRNA Array v3.0 (8×60 K; Arraystar), which was designed for 30,586 lncRNAs and 26,109 coding transcripts. The lncRNAs were carefully constructed using the most highly respected public transcriptome databases (RefSeq, UCSC Known Genes, GENCODE, etc.) as well as landmark publications. Each transcript was accurately identified by a specific exon or splice junction probe. Positive probes for housekeeping genes and negative probes were also printed onto the array for hybridization quality control. After washing the slides, the arrays were scanned using an Agilent G2505C scanner, and the acquired array images were analyzed with Agilent Feature Extraction software (version 11.0.1.1). Quantile normalization and subsequent data processing were performed using the GeneSpring GX v12.0 software package (Agilent Technologies). The microarray work was performed by KangChen Bio-tech, Shanghai, People’s Republic of China.

### Functional group analysis

GO analysis was derived from Gene Ontology (www.geneontology.org), which provides three structured networks of defined terms that describe gene product attributes. The P-value denotes the significance of GO Term enrichment in the differentially expressed mRNA list (P≤0.05 was considered statistically significant). We also performed pathway analysis for the differentially expressed mRNAs based on the latest KEGG (Kyoto Encyclopedia of Genes and Genomes) database. This analysis allowed us to determine the biological pathways for which a significant enrichment of differentially expressed mRNAs existed (P≤0.05 was considered statistically significant).

### Construction of the Coding-non-coding Gene Coexpression Network

The weighted coexpression network is constructed by calculating a pairwise correlation matrix between all probe sets across microarray samples. The resulting Pearson correlation matrix was transformed into an adjacency matrix. Microarray data can be noisy, and the number of samples is often small, so we weighed the Pearson.

correlations by taking their absolute value and raising them to the power. The nodes of coexpression network correspond to gene expressions, and edges between genes are determined by the correlations between gene expression Pearson correlation coefficients (Pearson correlation coefficient, PCC) was calculated between the coding and non-coding/coding and coding with the use of R statistical analysis. PCC> = 0.98 was considered statistically significant in both cases. The CNC (coding-non-coding)/CC (coding-coding) network was drawn with the Cytoscape (v2.8.1) software, which green nodes represent coding gene (mRNA), red nodes represent non-coding (lncRNA). The solid line represents a positive correlation between the two nodes, the dotted line represents a negative correlation.

### Quantitative PCR

Total RNA was extracted from frozen lung adenocarcinoma tissues by using TRIzol reagent (Invitrogen) and then reverse-transcribed using an RT Reagent Kit (Thermo Scientific), according to the manufacturer’s instructions. LncRNA expression in lung adenocarcinoma tissues was measured by quantitative PCR by using SYBR Premix Ex Taq and an ABI 7000 instrument. Some candidate lncRNAs were validated by SYBRP PCR, these gene primers in the study for Q-PCR see Table S3 in File S1. Among these, two lncRNAs that were significantly differentially expressed in adenocarcinoma and normal tissues (LOC100132354, RPLP0P2) were evaluated in all of the patients included in this study. Total RNA (2 mg) was transcribed to cDNA. PCR was performed in a total reaction volume of 20 µl, including 10 µl of SYBR Premix (2×), 2 µl of cDNA template, 1 µl of PCR forward primer (10 mM), 1 µl of PCR reverse primer (10 mM), and 6 µl of double-distilled water. The quantitative real-time PCR reaction included an initial denaturation step of 10 min at 95°C; 40 cycles of 5 s at 95°C, 30 s at 60°C; and a final extension step of 5 min at 72°C. All experiments were performed in triplicate, and all samples were normalized to GAPDH. The median in each triplicate was used to calculate relative lncRNA concentrations (△Ct = Ct median lncRNA − Ct median GAPDH), and the fold changes in expression were calculated [19].

### Statistical methods

All results are expressed as mean ± standard deviation. Statistical analysis was performed for the comparison of two groups in the microarray, and analysis of variance for multiple comparisons was performed using the Student’s *t*-test. Differences with P<0.05 were considered statistically significant in both cases. The fold change and the Student’s *t*-test were used to analyze the statistical significance of the microarray results. The false discovery rate (FDR) was calculated to correct the P-value. The threshold value used to designate differentially expressed lncRNAs and mRNAs was a fold change of ≥2.0 or ≤0.5 (P<0.05).

## Results

### Overview of lncRNA profiles

**Figure 1:**
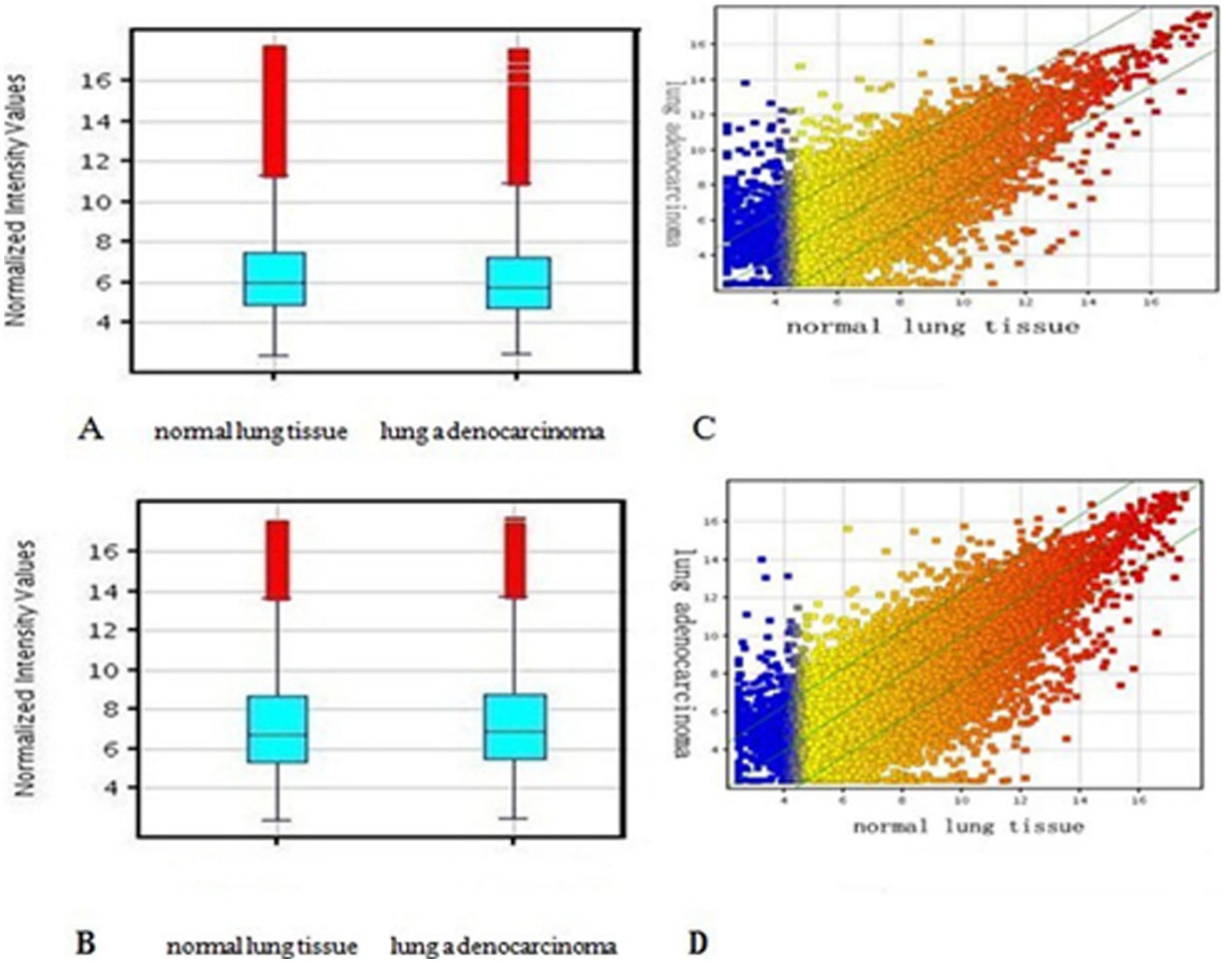
Box plots and Scatter plots showing the variation in lncRNA and mRNA expression between the lung adenocarcinoma and normal lung tissue arrays. (A–B) Box plots showing the distribution of the lncRNA (A) and mRNA (B) profiles. After normalization, the distributions of the log2-ratios among the tested samples were nearly the same. (C–D) Scatter plots showing the variation in lncRNA (C) and mRNA (D) expression between the lung adenocarcinoma and normal lung tissue arrays. The values of the X and Y axes in the scatter plot are averaged normalized values in each group (log2-scaled). The lncRNAs above the top green line and below the bottom green line are those with a >3-fold change in expression between tissues.

To study the potential biological functions of lncRNAs in lung adenocarcinoma, we examined the lncRNA and mRNA expression profiles in human lung adenocarcinoma through microarray analysis (Fig. 1). For this analysis, authoritative data sources containing more than 30,586 lncRNAs were used. The expression profiles of 2,420 lncRNAs indicated that they were differentially expressed (fold change ≥2.0 or ≤0.5; P<0.05) between lung adenocarcinoma and normal lung samples. Among these, 1,213 lncRNAs were found to be upregulated more than two-fold in the lung adenocarcinoma group compared to the normal lung group, while 1,207 lncRNAs were downregulated more than two-fold (P<0.05; Table S4, S5 in File S1 and Fig. 1).

### LncRNA classification and subgroup analysis

The expression profiles of 564 intergenic lncRNAs indicated that they were differentially expressed (fold change ≥2.0, P<0.05) between lung adenocarcinoma and normal lung samples. Among these, 338 were upregulated and 226 were downregulated. We also identified some nearby coding genes that may be regulated by these lncRNAs (Table S6 in File S1). LncRNAs with enhancer-like functions (lncRNA-a) were identified using GENCODE annotation. The expression profiles of 77 enhancer-like lncRNAs indicated that they were differentially expressed (fold change ≥2.0, P<0.05) between lung adenocarcinoma and normal lung samples. Among these, 43 were upregulated and 34 were downregulated. We also identified some nearby coding genes that may be regulated by these enhancer-like lncRNAs (Table S7 in File S1). HOX cluster profiling: This data table contains 83 HOX clusters (Table S8 in File S1).

### Overview of mRNA profiles

**Figure 2:**
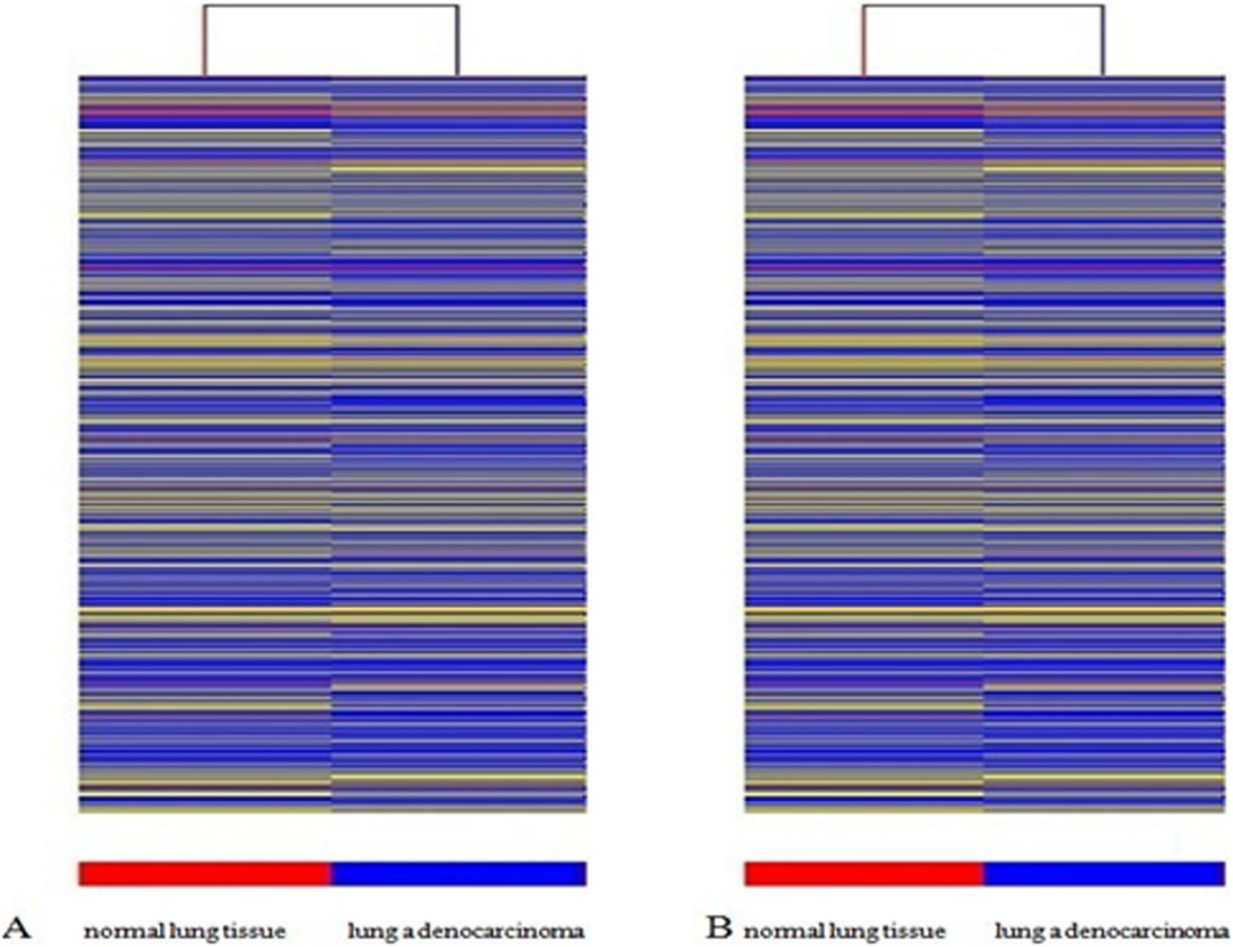
Heat map and hierarchical clustering of lncRNA and mRNA profile comparison between the lung adenocarcinoma and normal lung samples. (A) lncRNA (B) mRNA.

In total, 1,109 mRNAs were found to be differentially expressed between the lung adenocarcinoma and normal lung samples, including 278 that were upregulated and 831 that were downregulated (Tabel S9–S10 in File S1, Figs. 1–2).

### GO analysis

The genes corresponding to the downregulated mRNAs included 357 genes involved in biological processes, 98 genes involved in cellular components and 96 genes involved in molecular functions. The genes corresponding to the upregulated mRNAs included 244 genes involved in biological processes, 80 genes involved in cellular components, and 69 genes involved in molecular functions(Tabel S11–S12 in File S1).

### Pathway analysis

**Figure 3:**
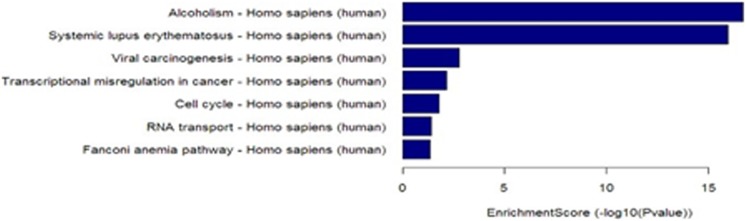
Pathway analysis of upregulated mRNAs in lung adenocarcinoma. Seven upregulated pathways were identified, including ethanol metabolism, viral carcinogenesis, RNA transduction, and cell cycle pathways.

**Figure 4:**
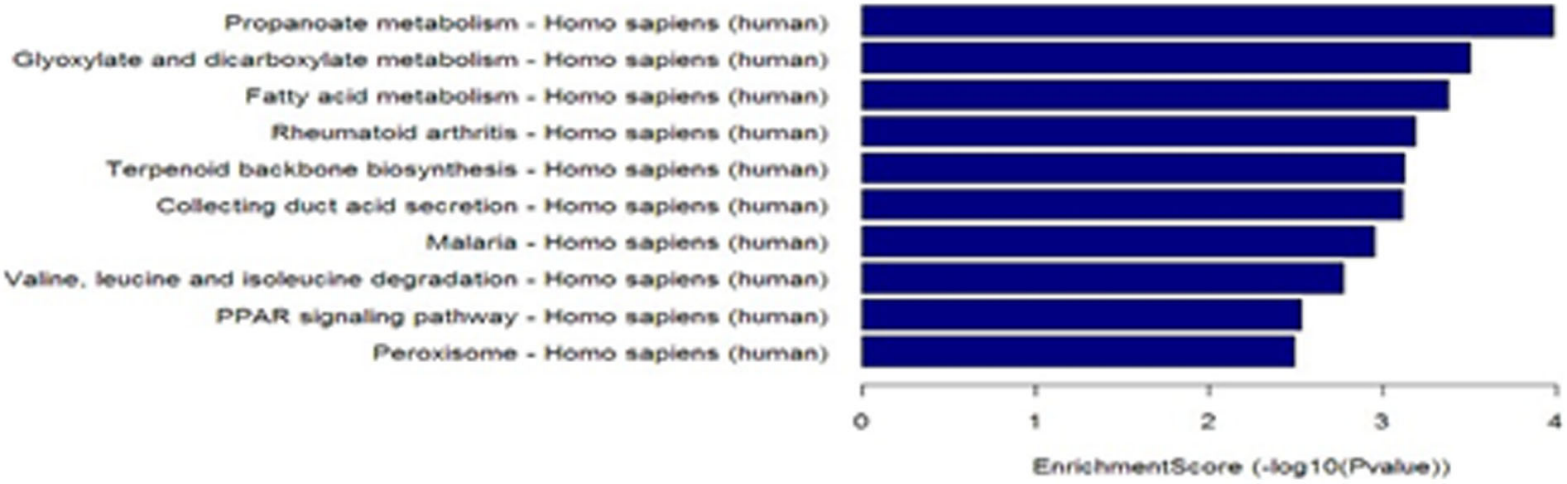
Pathway analysis of downregulated mRNAs in lung adenocarcinoma. Twenty-four downregulated pathways were identified, including propionate metabolism and fatty acid metabolism pathways.

Seven upregulated pathways were identified, including ethanol metabolism, viral carcinogenesis, RNA transduction, and cell cycle pathways. Twenty-four downregulated pathways were identified, including propionate metabolism and fatty acid metabolism pathways (Tables S13 in File S1and figure 3–4).

### LncRNA-mRNA co-expression network

We build the LncRNA-mRNA co-expression network of four important lncRNAs, including RP11-1C1.7 (ENST00000416930), RPLP0P2 (ENST00000492786), LOC100132354 (NR_024478), RP11-909N17.3 (ENST00000519762), See Table S14 in File S1.

### Real-time quantitative PCR validation

**Figure 5:**
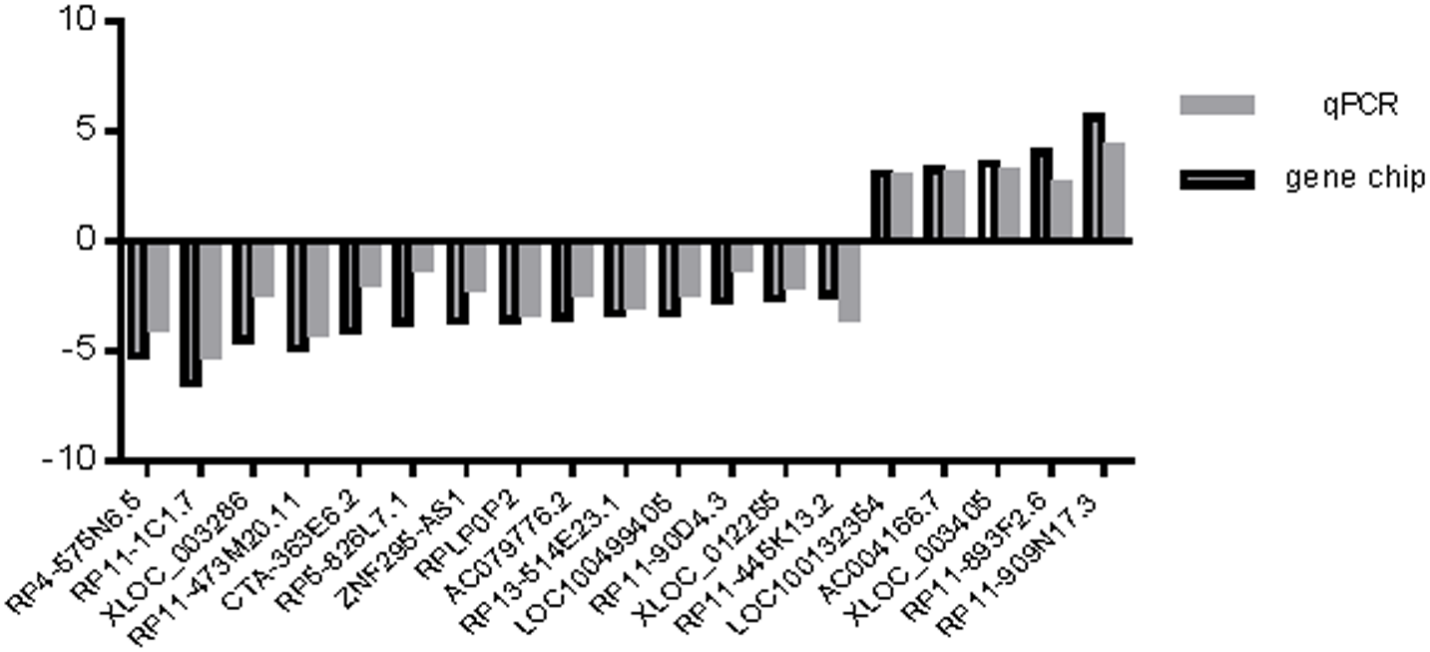
Comparison between gene chip data and qPCR result. RP11-1C1.7, RP4-575N6.5, RP11-473M20.11, XLOC_003286, CTA-363E6.2, RP5-826L7.1, ZNF295-AS1, RPLP0P2, AC079776.2, RP13-514E23.1, LOC100499405, RP1-90D4.3, XLOC_012255, RP11-445K13.2, LOC100132354, AC004166.7, XLOC_003405, RP11-893F2.6, RP11-909N17.3 determined to be differentially expressed in lung adenocarcinoma samples compared with NT samples in six patients by microarray were validated by qPCR. The heights of the columns in the chart represent the log-transformed median fold changes (T/N) in expression across the six patients for each of the four lncRNAs validated; the bars represent standard errors. The validation results of the 19 lncRNAs indicated that the microarray data correlated well with the qPCR results.

**Figure 6:**
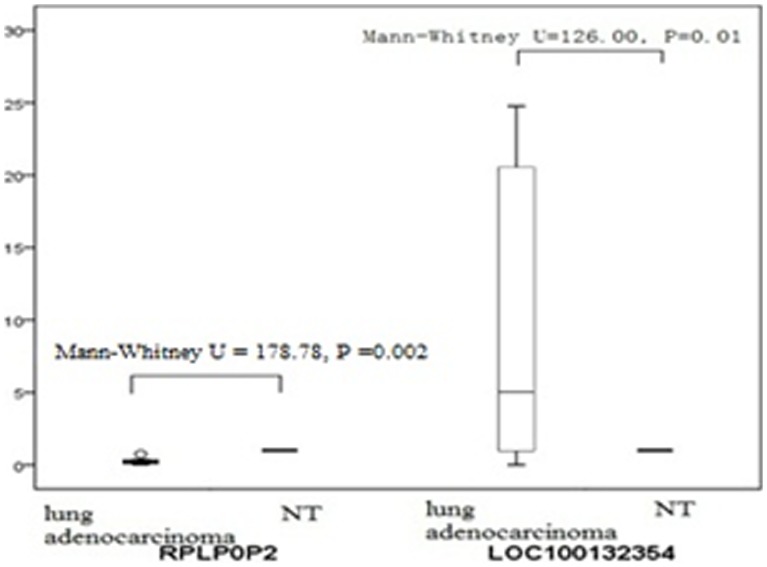
The expression level of RPLPOP2 and LOC100132354 between one hundred lung adenocarcinoma sample and the normal lung sample. LOC100132354 expression of lung adenocarcinoma was significantly higher than the adjacent tissues (Mann-Whitney U = 126.00, P = 0.01), while RPLP0P2 expression of lung adenocarcinoma was significantly lower than the adjacent tissues (Mann-Whitney U = 178.78, P = 0.002).

Based on the features of the differentially expressed lncRNAs, such as fold difference, gene locus, and nearby encoding gene, we initially identified a number of interesting candidate lncRNAs for further analysis (including RP11-1C1.7, RP4-575N6.5, RP11-473M20.11, XLOC_003286, CTA-363E6.2, RP5-826L7.1, ZNF295-AS1, RPLP0P2, AC079776.2, RP13-514E23.1, LOC100499405, RP11-90D4.3, XLOC_012255, RP11-445K13.2, LOC100132354, AC004166.7, XLOC_003405, RP11-893F2.6, and RP11-909N17.3). We verified the expression of these lncRNAs by real-time quantitative RT-PCR by using GAPDH as a reference gene and by calculating the 2^−ΔΔCT^ values. We found that the microarray results for several of the lncRNAs were consistent with the results of RT-PCR (Fig. 5). Of these, LOC100132354 and RPLP0P2 exhibited the most significantly changed expression in our analysis of 100 pairs of lung adenocarcinoma and normal lung tissue samples. The expression of LOC100132354 was significantly higher in lung adenocarcinoma than in the adjacent tissues (Mann-Whitney U = 126.00, P = 0.01), while the expression of RPLP0P2 was significantly lower in lung adenocarcinoma than in the adjacent tissues (Mann-Whitney U = 178.78, P = 0.002) (Fig. 6).

## Discussion

According to the 2012 China Oncology Annual Report, in 2009, the incidence and mortality of lung cancer was the highest among cancers in male patients and the second highest among cancers in female patients in China. Lung adenocarcinoma is a type of NSCLC that has rising incidence rates in women and non-smokers. However, the pathogenesis of lung cancer remains unclear; therefore, further study of lung cancer is of great importance.

LncRNAs play an important role in many biological processes, including X-chromosome inactivation, gene imprinting, and stem cell maintenance [20], [21]. Furthermore, lncRNAs are important factors in the control of gene expression in cancer [22], and lncRNAs such as HOTAIR have been shown to play an important role in the development and progression of tumors [9]. It has also been demonstrated that lncRNAs are differentially expressed in normal cells and tumor cells. As lncRNAs constitute an important class of gene expression regulatory factors, their aberrant expression would inevitably lead to abnormal gene expression levels, which may result in tumorigenesis. Promoters bind to many transcription factors by mechanisms such as chromosomal rearrangements and transfer elements [23].

In this study, we analyzed lncRNA expression profiles in the tissues of lung adenocarcinoma patients to uncover the potential role of lncRNAs in the pathogenesis of this disease. High-throughput microarray techniques revealed a set of differentially expressed lncRNAs, including 1,213 that were upregulated and 1,207 that were downregulated in lung adenocarcinoma tissue compared to normal lung tissue. LncRNAs are usually divided into five categories: sense, antisense, bidirectional, intronic, and intergenic. LncRNAs are known to function via a variety of mechanisms; however, a common and important function of lncRNAs is to alter the expression of nearby encoding genes by affecting the process of transcription [24] or directly playing an enhancer-like role [25], [26]. In the present study, we increased the accuracy of target prediction by comparing differentially expressed mRNAs with differentially expressed lncRNAs. The lncRNA expression profiles indicated that 564 lncRNAs were differentially expressed (338 upregulated and 226 downregulated) between lung adenocarcinoma and normal lung samples. The expression profiles included 77 differentially expressed enhancer-like lncRNAs, with 43 upregulated and 34 downregulated. We also identified some nearby coding genes that may be regulated by lncRNAs and enhancer-like lncRNAs. Moreover, we also performed HOX cluster profiling of lncRNAs and coding transcripts.

Otherwise,we build the LncRNA-mRNA co-expression network and we analyzed four important lncRNAs, including RP11-1C1.7, RPLP0P2, LOC100132354, RP11-909N17.3. It shown that RP11-1C1.7, RPLP0P2, LOC100132354, RP11-909N17.3 were individually relation to some mRNAs.

In order to obtain insights into lncRNA target gene function, GO analysis and KEGG pathway annotation were applied to the lncRNA target gene pool. GO analysis revealed that the number of genes corresponding to downregulated mRNAs was larger than that corresponding to upregulated mRNAs. KEGG annotation showed that there were seven upregulated pathways (including ethanol metabolism, viral carcinogenesis, RNA transduction, and cell cycle pathways) and 24 downregulated pathways (including propionate metabolism and fatty acid metabolism pathways). These pathways might play important roles in the occurrence and development of lung adenocarcinoma. We found that 19 of the lncRNAs identified in the microarray analysis were confirmed by RT-PCR to be aberrantly expressed in lung cancer tissues. Among these lncRNAs, LOC100132354 was the most significantly upregulated and RPLP0P2 was the most significantly downregulated. This result suggests that LOC100132354 and RPLP0P2 might contribute to the development of lung adenocarcinoma; further study of the biological function of LOC100132354 and RPLP0P2 will be required to confirm this notion.

To summarize, our study revealed a set of lncRNAs with differential expression in lung adenocarcinoma compared with normal lung tissue. Furthermore, potential roles for these lncRNAs in the regulation of ethanol metabolism and propionate metabolism signaling pathways were identified. Moreover, we found that LOC100132354 and RPLP0P2 might contribute to the development of lung adenocarcinoma. Further investigation of the lncRNAs identified in this study will likely shed light on their biological functions and their association with lung cancer.

## Supporting Information

File S1 **Contains the files: Table S1**, The demographics characteristics of 5 cases lung adenocarcinoma for microarray analysis. **Table S2**, The demographics characteristics of 100 cases lung adenocarcinoma. **Table S3**, LncRNAs gene primers in the study for Q-PCR. **Table S4**, Upregulated lncRNAs in lung adenocarcinoma. **Table S5**, Downregulated lncRNAs in lung adenocarcinoma. **Table S6**, LincRNAs profile of lung adenocarcinaoma. **Table S7**, Enhaner lncRNAs profile of lung adenocarcinaoma. **Table S8**, HOX profile of lung adenocarcinaoma. **Table S9**, Upregulated mRNA in lung adenocarcinoma. **Table S10**, Downregulated mRNA in lung adenocarcinoma. **Table S11**, GO analysis upregulated mRNAs. **Table S12**, GO analysis downregulated mRNAs. **Table S13**, Pathway analysis. **Table S14**, CNC_network.
(RAR)
